# Case report: Blotchy skin in a puffy neonate: is there a new association?

**DOI:** 10.3389/fped.2023.1247343

**Published:** 2023-09-21

**Authors:** Chacko J. Joseph, Arijit Lodha, Soumya R. Thomas, Essa Al Awad, Nicola A. M. Wright, Cora Constantinescu, Doan Le, Majeeda Kamaluddeen

**Affiliations:** ^1^ Section of Neonatology, Department of Pediatrics, Cumming School of Medicine, University of Calgary, Calgary, AB, Canada; ^2^ Alberta Health Services, Calgary, AB, Canada; ^3^ Faculty of Medicine & Dentistry, University of Alberta, Edmonton, AB, Canada; ^4^ Alberta Children’s Hospital Research Institute, Cumming School of Medicine, University of Calgary, Calgary, AB, Canada; ^5^ Section of Infectious Diseases, Department of Pediatrics, Cumming School of Medicine, University of Calgary, Calgary, AB, Canada

**Keywords:** purpura fulminans, chylothorax, congenital, newborn, pleural effusion

## Abstract

**Introduction:**

Purpura fulminans in the neonatal population is a rare but potentially life-threatening condition complicated by thrombosis, resultant vital organ necrosis, and gangrene of the extremities. Considering the rapid evolution of the pathogenetic mechanism, an index of suspicion, early identification, and prompt intervention are imperative for improved outcomes. The majority of purpura fulminans cases have an infectious etiology, but it is essential to consider other congenital and acquired causes.

**Case description:**

We present a clinical case of a female neonate to emphasize the correlation between purpura fulminans, congenital chylothorax, involvement of the *PAK2* gene, and the occurrence of retinal detachment in both eyes. After draining the congenital chylothorax, the neonate developed purpura fulminans due to a loss of protein C, S, and antithrombin factors, previously not reported in the literature. The purpuric lesions resolved after the administration of fresh frozen plasma. Subsequently, no recurring purpura fulminans lesions were noted following the normalization of the antithrombotic factor levels in the serum. Subsequently, the child also developed retinal detachment in both eyes.

## Introduction

Pleural effusion in the fetus is a rare but serious finding on antenatal ultrasound screening. Most cases of congenital pleural effusion are associated with other findings such as hydrops, chylothorax related to lymphatic malformations, significant cardiac malformations, and genetic syndromes like trisomies. A smaller proportion of cases may be isolated with no other associations or a confirmed etiology for effusion; these are referred to as primary pleural effusion (PPE). The prognosis of fetal PPE is variable, with reports suggesting that almost half remain stable or spontaneously resolve with increasing gestation while the remaining slowly progress. The progressive group has a higher incidence of fetal interventions and poorer perinatal outcomes (1). Outcomes in PPE tend to be more favorable as the neonatal survival range is 70%–90% (1, 2). A pleural effusion that occurs secondary to malformations has a variable prognosis, and a chylothorax associated with lymphoproliferative disorders tends to have a poor prognosis.

The neonatal course of infants with congenital chylothorax is often complicated with cardiorespiratory failure, need for thoracocentesis, immune dysfunction, slow initiation of feeds, interruptions in feed advancements, re-accumulation of fluid, and malnutrition due to the ongoing loss of protein-rich pleural fluid. Purpura fulminans (PF) has not been reported in neonates with chylothorax. In this first case report, we describe a term newborn presenting with congenital chylothorax associated with PF, who showed symptoms on the first post-natal day, shortly after the drainage of the chylothorax. Also, this case focuses on the possible underlying mechanisms of developing PF in congenital chylothorax, early recognition, management, and having a high index of suspicion for early detection.

## Case report

A female infant with 37 weeks’ gestation of a birth weight of 2.775 kg (appropriate for gestational age) was delivered by cesarean section for breech presentation and Hemolysis, Elevated Liver enzymes and Low Platelets (HELLP) syndrome. The mother was a healthy gravida two, para one with no history of consanguinity. The mother did not have a history of lupus erythematosus. The previous child was healthy and had no history of bleeding disorders at birth and there was no family history of thrombosis. Also, there were no concerns of trauma during birth for the neonate. The mother's blood group was O, Rh-negative, with a positive antibody screen after anti-D administration and there were no features of fetal anemia. Antenatal serological tests for toxoplasma, cytomegalovirus, and parvovirus were negative. In addition, there was no evidence of a COVID-19 infection in the mother during pregnancy or at the time of delivery. There was no maternal history of fever or prolonged rupture of membranes. However, at 20 weeks gestation, antenatal scans showed persistent bilateral pleural effusion in the fetus, which was larger on the left side. No other fluid collections or malformations were noted. Rapid aneuploidy analysis showed negative results for Trisomy 21 and Monosomy X. Antenatal pleural fluid aspiration showed 95% lymphocytes, suggesting chylothorax.

At birth, the neonate had no respiratory effort with bradycardia, which improved following mask intermittent positive pressure ventilation (IPPV). The Apgar score at 1 and 5 min were 3 and 7, respectively. Subsequently, the neonate was intubated in the delivery room due to an increase in work of breathing and decreased air entry bilaterally. Thoracocentesis with aspiration of 10 and 49 ml of serous fluid from the right and left pleural spaces, respectively, was performed upon admission to the neonatal intensive care unit (NICU) due to the neonate's severe respiratory distress, needing high-frequency oscillatory ventilator support. After birth, the fluid that was obtained by thoracocentesis was cloudy and slight yellow in color. The pleural fluid was analyzed for biochemistry and cell counts, revealing a total white blood cell count of 1,364 (comprising 93% lymphocytes and 3% macrophages), lactate dehydrogenase (LDH) level of 78, and fluid triglycerides measuring <0.10. These findings suggested the possibility of chylothorax, even in the absence of milk or formula feeding. Subsequently, cloudy fluid volumes of 96 and 15 ml were drained through the right and left chest tubes, respectively. The fluid analysis satisfied Light's criteria for exudate based on elevated LDH, and the predominance of lymphocytes confirmed the diagnosis of chylothorax. However, we did not measure coagulation factors in the pleural fluid, as this is not a routine practice in the NICU. In addition, there are no established normal values for these factors in pleural fluids. Repeat chest x-rays (CXR) after fluid drainage revealed bilateral pneumothoraces that were drained by needle thoracocentesis. Later on, the neonate developed further increased work of breathing due to tension pneumothorax leading to hemodynamic instability that required repeated bilateral needle thoracentesis, followed by bilateral chest tube insertion and fluid resuscitation. On oscillatory ventilation, the neonate developed a higher oxygen requirement with persistent anterior pneumothorax, which warranted a transition to jet ventilation and improved oxygenation and ventilation. The duration of low pH, hypoxia, and acidosis lasted for only 2–3 h.

The first echocardiographic assessment of the heart showed a small ventricular septal defect, patent ductus arteriosus, with evidence of pulmonary hypertension for which inhaled nitric oxide was commenced.

**Figure 1:**
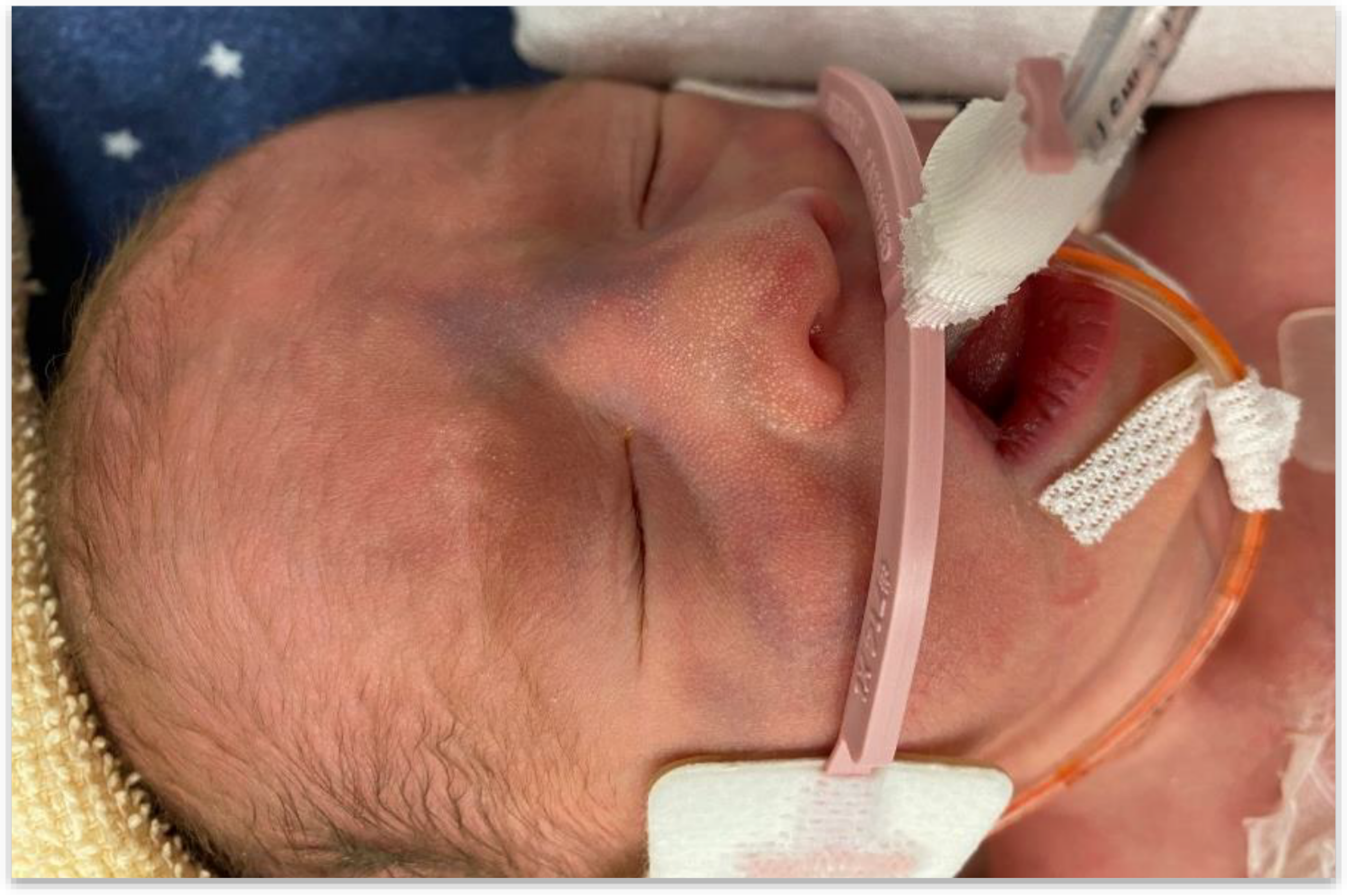
Purpuric lesions over the face.

**Figure 2:**
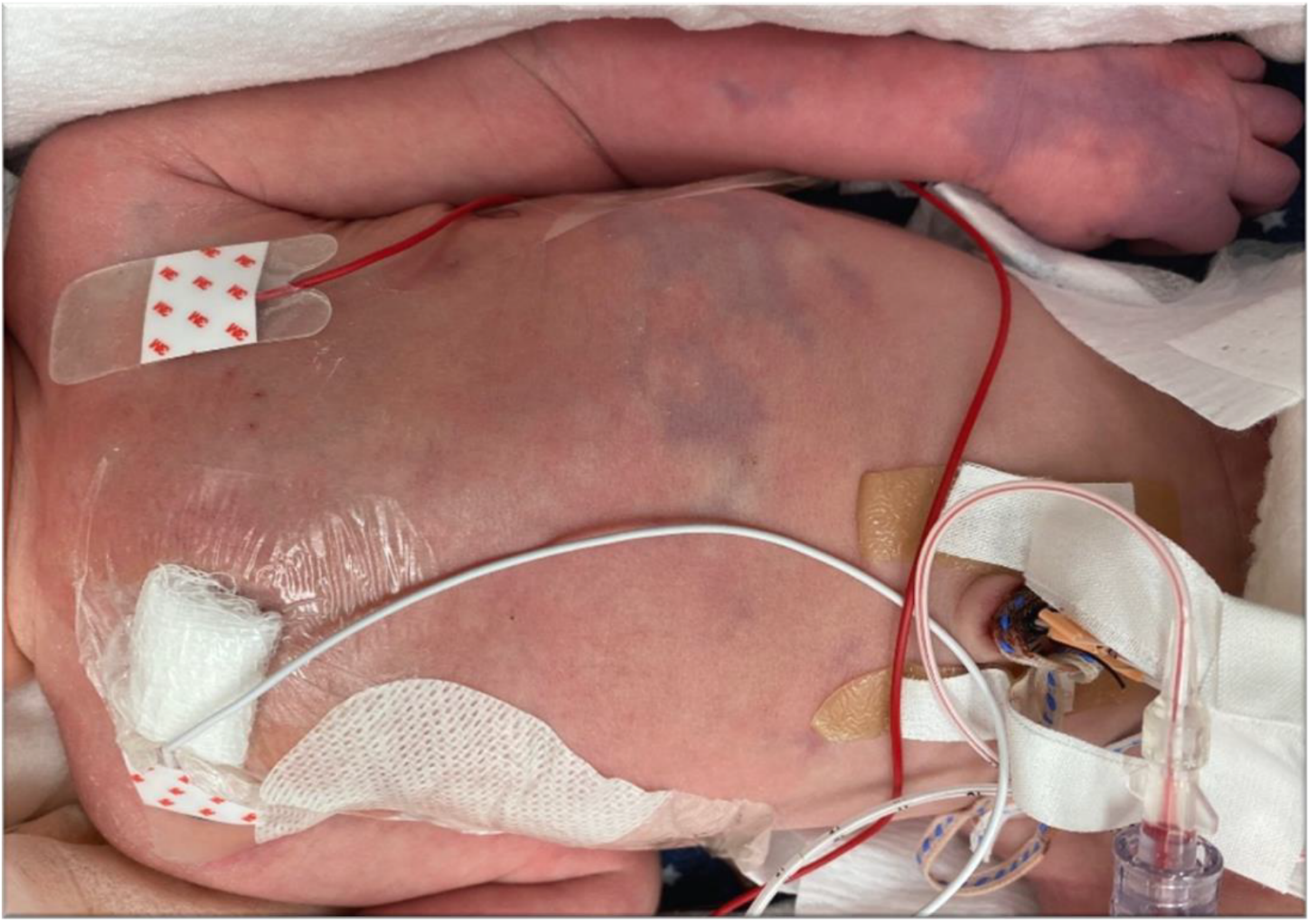
Purpuric lesions over the abdomen and the arm.

After chest drain and aspiration of chylous fluid, at 12 h of age, the neonate acutely developed well-demarcated erythematous macules that progressed rapidly to develop irregular central areas of blue-black purpuric lesions over the face, abdomen, and arms (Figures 1, 2). The lesions did not blanch with digital pressure application, and no separate petechial lesions or any other signs of bleeding were evident. All septic markers, including cell blood counts and C-reactive protein, were within normal limits. Due to the purpuric lesions, infectious diseases, hematology, and dermatology specialties were consulted, blood cultures were repeated, and the infection screen was expanded to include chlamydia, herpes and fungal cultures, and herpes blood PCR. Antibiotics were changed from first-line ampicillin and gentamicin to vancomycin, meropenem, and clindamycin. Prothrombin time (PT), international normalized ratio (INR), and activated partial thromboplastin time (aPTT) assays, protein C, S antigen, fibrinogen, antithrombin (AT) and D-dimer levels, and protein S activity assay were performed before and after transfusion of fresh frozen plasma (FFP) (Table 1). The International Society for Thrombosis and Haemostasis (ISTH) disseminated intravascular coagulation (DIC) score was 7, which indicates that the neonate had laboratory evidence consistent with overt DIC related to purpura fulminans. Since purpura fulminans did not persist for a long time, he did not require any anticoagulant agent.

**Table 1:** Coagulation tests and anticoagulant profile.

Assay	17 h	20 h	48 h	102 h
PT	20.1 s	—	18.3 s	16.0 s
INR	1.7	1.9	1.6	1.4
aPTT	75.3 s	68.2 s	61.3 s	58.7
Fibrinogen level (g/dl)	1.21	—	—	—
Protein C (U/ml)	0.07	0.11	0.09	
Protein S antigen (U/ml)	0.27	—	—	—
Protein S activity (U/ml)	0.21	—	—	—
AT (U/ml)	0.15	—	0.19	0.14
D-dimer (mg/L)	—	4.45	—	—

The complete blood counts were sent at the time of onset of the skin lesions and after disappearance of PF (Table 2). The CBC results indicated a normal count within the first 24 h of life; however, after 24 h of life, there was a drop in platelet counts (as shown in Table 2). Serum lactate levels remained normal during this period. Blood cultures; blood PCR for herpesvirus 1, 2, and varicella-zoster; surface swab PCR for herpes virus; surface swab fungal culture; and pleural fluid culture reports were negative. The coagulation profile test results have been detailed below, with timelines (Table 1).

**Table 2:** Complete blood count profiles.

	1 h	10 h	16 h	25 h	43 h	53 h	77 h	92 h	150 h
Hemoglobin	191	189	181	150	185	152	163	161	144
Hematocrit	0.56	0.55	0.53	0.46	0.53	0.44	0.48	0.47	0.40
Platelet counts	226	178	188	95	41	38	42	54	177
WBC	11.6	19.1	16.8	15.7	12.4	9.6	9.8	10.4	13.4

The low levels of protein C, antithrombin, and borderline protein S on day 1 of life may have been a result of the loss of these factors in the pleural fluid. There was no history of prolonged hypoxia or acidosis. These low levels resolved with FFP transfusions. FFP was administered every 12 h on day 1 of life, followed by one transfusion on day 2, discontinued on day 3 after typical coagulation results, and remained normal on day 4. Sepsis work-up results were negative, and we could not rule out any other possible cause of disseminated intravascular coagulopathy. The platelet count remained within normal limits at the onset of purpura fulminans (day 1); however, platelet levels subsequently dropped from day 2 to day 5 of life, indicating possible evidence of DIC (as shown in Table 2). The cause of DIC and low factor assay remained unknown. The elevated D-dimer assay was attributed to the predisposition to thrombophilia due to the loss of the antithrombotic factors after draining the chylothorax fluid. The purpura resolved following FFP transfusion with no further recurrence. The baby continued to have intermittent skin mottling episodes, with the appearance of cutis marmorata, which resolved spontaneously without intervention. As part of genetic work-up, RAPID exome sequencing was performed. The clinical exome report, however, did report a variant of unknown clinical significance in a gene called *PAK2* without any clinical significance. This specific variant is c.1115A > T, p.Asp732Val. Furthermore, the results did not reveal any variants or mutations in the *PROC*, *PROS*, and *SERPINC1* genes.

The child was referred to an ophthalmologist for an eye examination due to the absence of a red reflex at the time of discharge. The ophthalmologist performed a thorough assessment using dilated fundus examination and conducted intravenous fluorescein angiography along with B-scan ultrasound. The B-scan ultrasound confirmed retinal detachments in both eyes but ruled out retinoblastoma, congenital cataract, or calcifications. Fluorescein angiography revealed a funnel retinal detachment in the right eye and a knife-edge detachment in the left eye. Furthermore, retinal vessels were identified between 4 o'clock and 7 o'clock in the left eye, along with a small avascular crescent of retina at the far periphery in this clock hour. However, there was no neovascularization observed in either eye.

## Discussion

Purpura fulminans in the newborn period is a rare condition. The etiological causes of PF in newborns can be varied. Acute infections with *Neisseria meningitidis*, *Group A Streptococci*, Gram-negative bacilli, *varicella-zoster* infection, and rarely *Staphylococcus aureus* have been implicated (3–5). Noninfectious acquired conditions predisposing to PF include acute venous thrombosis, antiphospholipid antibodies, cardiac bypass, severe hepatic dysfunction, and the use of warfarin therapy. Among the congenital conditions, in addition to homozygous protein C and S deficiency, neonates with galactosemia and congenital heart disease are at risk of developing purpura fulminans.

Both congenital and acquired protein C and S deficiency can result in PF due to a predisposition to thrombophilia (6, 7). Congenital protein C and S deficiencies occur as autosomal dominant conditions with varied penetrance and widely varying clinical presentations (8). Both homozygous protein C and S deficiency genotypes are associated with neonatal purpura fulminans. The diagnosis is based on the clinical presentation of PF with deficient protein C or S activity levels and molecular genetic testing to confirm parental genotype. Protein C synthesized in the liver depends on vitamin K's availability for carboxylation. Activation of the protein involves forming a complex with thrombin followed by the complex binding to the endothelial cell surface receptors like thrombomodulin (TM) and endothelial protein C receptor (EPCR). Activated protein C (APC) exerts its antithrombotic effects by regulating proteolysis and activating factors V and VIII. Protein S enhances the activity of APC. A reduction in either of these anticoagulation proteins reduces the ability to regulate thrombin generation, leading to hypercoagulability.

The primary pathology in PF includes microvascular thrombosis of the dermal vasculature with resultant perivascular hemorrhage. The thrombotic event is associated with DIC. The severity of clinical presentation is variable, depending on the underlying cause. Neonates with homozygous deficiency usually present with rapidly developing purpuric lesions on the skin after birth within the first 2–12 h of life. A rare form of the condition has been reported, which presents between 6 and 12 months. Purpura fulminans associated with infections also tend to progress rapidly with the risk of complications. The lesions show a predilection for the limbs, although the thighs and buttocks may also be affected. Previous sites of trauma (intravenous cannula insertion sites) are often preferentially affected. The initial dark red lesions turn into indurated purple-black lesions. A delay in identifying and managing the condition could result in necrosis and gangrene of the extremities. Other complications associated with the underlying thrombotic process include retinal vein, artery or vitreal vein thrombosis resulting in retinal detachment, vitreous hemorrhage, blindness, cerebral vein thrombosis, ischemic stroke, hydrocephaly, arterial and arterial intracardiac thromboembolic manifestations (9–12). Hence, early diagnosis and effective management of neonatal PF are essential.

When presented with PF, the distinction between acquired and congenital etiologies can be challenging, considering that laboratory findings include thrombocytopenia, elevated PT, activated partial thromboplastin time (aPTT), increased fibrin degradation products (FDP), hypofibrinogenemia, and decreased activity levels of protein C, S, and antithrombin may occur in both etiological groups. Age-appropriate reference ranges must be used to interpret coagulation factor activity levels since factor levels in healthy neonates (0.12–0.14 U/ml) tend to be significantly lower than in adults. However, the levels may be undetectable in homozygous newborns. Molecular genetic testing of the neonate and parents would help in diagnostic confirmation but may not be timely enough to assist in the acute management of the condition.

Due to deficiency of coagulation factors (congenital or acquired), the primary objective of management in neonatal PF is to restore the serum levels of the antithrombotic factors, including protein C and S. Fresh frozen plasma transfusion and plasma-derived protein C concentrate are available. Following up on the coagulation factor levels is recommended once the levels have normalized in transient deficiency cases. In congenital deficiency of coagulation factors, long term anticoagulants and FFP are recommended, or the use of factor C concentrate during acute thrombotic breakthrough events.

Congenital chylothorax is the most common cause of congenital pleural effusion in the fetus and during the neonatal period (13, 14). Close to 65% of fetal hydrothorax are diagnosed as chylothorax. The estimated frequency of chylothorax can range from one in every 10,000 to 24,000 births (13, 15). The condition occurs twice as commonly in male fetuses compared to females and is more common on the right side than on the left (14). The diagnosis of chylothorax is confirmed by pleural fluid analysis, *in utero* or after birth. While neonates are not fed, the fluid color remains clear, without a milky appearance and without showing elevated levels of triglycerides. However, the fluid does contain more than 1,000 white blood cells per ml, of which >90% are lymphocytes. In addition, there is a significant protein content. With the initiation of feeding, the fluid consistency turns creamy, and a triglyceride concentration of >1,000 mg/dl of pleural fluid is seen. Large chylothorax and its subsequent drainage are associated with decreased plasma antithrombin activity and the loss of lymphocytes, antibodies, and complement and coagulation factors. These predispose the neonate to a higher risk of hospital-acquired infections and bleeding (16, 17). In this case, we encountered drainage of the chylothorax, followed by the development of purpura fulminans. The activity assays of both protein C and S were low (for age-appropriate reference ranges) when signs of PF and skin manifestations developed. However, when FFP was transfused, skin manifestations resolved and levels of coagulation factors improved, and no future recurrence was reported.

In our case, we observed the development of PF after the drainage of pleural effusions. This development could possibly be attributed to the loss of coagulation factors in the fluids. Notably, this chylothorax was present at birth and did not emerge after surgical injury to the thoracic ducts, indicating a congenital origin. The fact that this chylothorax did not recur and was resolved suggests the absence of evidence for thoracic duct obstruction. In terms of genetic work-up, until now, *PAK2* has not been definitively associated with any human diseases, including purpura fulminans. The results also did not find any mutations in the *PROC*, *PROS*, and *SERPINC1* genes, ruling out congenital natural anticoagulant deficiencies. However, concerning the ocular changes characterized by retinal detachment, there are several potential explanations. It could be associated with thrombosis linked to PF, or it might be related to conditions such as familial exudative vitreoretinopathy (FEVR), an extreme phenotype of persistent fetal vasculature, or the rare Stickler syndrome. Our ophthalmologist has ruled out the likelihood of Stickler syndrome in our case. The presence of FEVR is a possibility; this disease is characterized by abnormal retinal angiogenesis, leading to incomplete vascularization of the peripheral retina, which in turn can result in retinal ischemia and detachment.

## Conclusion

In conclusion, this case sheds light on the development of neonatal purpura fulminans subsequent to the drainage of a significant congenital chylothorax. This occurrence appears to be linked to a transient deficiency of protein C and S, likely arising from the loss of coagulation factors and *PAK2* gene variant. In addition, the presence of retinal detachments in this case, an unprecedented finding in the literature, adds to the complexity.

Given the urgency associated with neonatal purpura fulminans, it becomes imperative to recognize its potential connection with chylothorax drainage. This necessitates vigilant monitoring of serum coagulation factor levels, prophylactic replacement of chylous fluid with fresh frozen plasma, and consideration of genetic testing for a range of associated genes. Furthermore, the inclusion of emergent ophthalmological examinations should be an integral part of the treatment strategy.

## Data Availability

The original contributions presented in the study are included in the article/Supplementary Material, further inquiries can be directed to the corresponding author.
